# Career planning for specialist medical training through network structures: A successful model in times of medical workforce shortages?

**DOI:** 10.3205/zma001817

**Published:** 2026-02-17

**Authors:** Mirjam Thanner, Rahel Meyer, Ellie B. Schmidt, René Hornung

**Affiliations:** 1 Cantonal Hospital St. Gallen, Women's Clinic, St. Gallen, Switzerland

**Keywords:** medical training, medical specialist, network structures, network, gynaecology and obstetrics

## Abstract

**Introduction:**

Alliance structures with regional networks of various hospitals and medical practices should improve specialist medical training. While official training institutions in Switzerland must be embedded in network structures, young physicians seeking to specialise are free to choose a specific network and complete all stages of their training within that network, or to combine training at training institutions from different networks.

**Objective:**

The aim was to use the application and career paths of junior doctors to describe how a network can be implemented in the context of specialist medical training and to draw conclusions for practice.

**Method:**

All applications received between 1 January 2012 and 31 December 2022 at the Women's Clinic of the Cantonal Hospital of St. Gallen in Switzerland for a position as a junior doctor pursuing specialist training in the field of gynaecology and obstetrics were reviewed, coded according to predefined questions and then quantitatively analysed. The research questions included sociodemographic characteristics, level of training at the time of application, career path within the network and the acquisition of specialist titles.

**Results:**

A total of 415 applications were analysed. There were 336 female applicants (81.0 percent) and 79 male applicants (19.0 percent). Overall, the applicants held medical degrees from 37 different countries. The proportion of applicants with foreign degrees was 91.6 percent. The majority (78.8 percent) of applications were processed with reference to the entire training network. Only about one in five applications (21.2 percent) was processed independently of the network.

**Conclusions:**

The applications for junior doctor positions at the hospital described are characterised by international diversity. Network structures increase the reach of applications and can support the participating training institutions in recruiting medical staff. This requires the willingness of the participating institutions to invest in networking.

## Zusammenfassung

**Einführung:**

Verbundstrukturen sollen als regionale Netzwerke von verschiedenen Spitälern und Praxen die fachärztliche Weiterbildung verbessern. Während offizielle Weiterbildungsstätten in der Schweiz in Verbundstrukturen eingebettet sein müssen, steht es dem fachärztlichen Nachwuchs frei, sich bewusst für ein bestimmtes Netzwerk zu entscheiden und alle Weiterbildungsabschnitte in diesem Netzwerk zu durchlaufen oder einzelne Weiterbildungsstätten unterschiedlicher Netzwerke zu kombinieren.

**Zielsetzung:**

Ziel war es, anhand der Bewerbungs- und Karriereverläufe von Assistenzärzt:innen die Arbeitsweise eines Netzwerkes im Rahmen der ärztlichen Weiterbildung exemplarisch zu beschreiben und Schlussfolgerungen für die Praxis abzuleiten.

**Methode:**

Alle im Zeitraum vom 01.01.2012 bis 31.12.2022 an der Frauenklinik des Kantonsspitals St. Gallen in der Schweiz eingegangenen Bewerbungen für eine Anstellung als Assistenzärzt:in zur Weiterbildung im Fachgebiet Gynäkologie und Geburtshilfe wurden gesichtet, hinsichtlich der vorher festgelegten Fragestellungen kodiert und im Anschluss quantitativ ausgewertet. Die Forschungsfragen umfassten u.a. soziodemographische Merkmale, Weiterbildungsstand zum Zeitpunkt der Bewerbung, Karrierepfad im Netzwerk und fachärztlichen Titelerwerb.

**Ergebnisse:**

Insgesamt wurden 415 Bewerbungen analysiert. Es bewarben sich 336 Frauen (81.0 Prozent) und 79 Männer (19.0 Prozent). Insgesamt besaßen die Bewerber:innen ärztliche Diplome aus 37 verschiedenen Ländern. Der Anteil an Bewerber:innen mit ausländischem Diplom lag bei 91.6 Prozent. Ein Großteil (78.8 Prozent) der Bewerbungen ließ sich im Sinne des gesamten Weiterbildungsnetzwerkes nutzen. Nur etwa jede fünfte Bewerbung (21.2 Prozent) wurde unabhängig vom Netzwerk bearbeitet.

**Schlussfolgerungen:**

Die assistenzärztlichen Bewerbungen am beschriebenen Krankenhaus zeichnen sich durch internationale Vielfalt aus. Verbundstrukturen vergrößern die Reichweite von Bewerbungen und können die beteiligten Weiterbildungsstätten bei der Rekrutierung von ärztlichem Personal unterstützen. Voraussetzung dafür ist die Bereitschaft der beteiligten Institutionen, in Netzwerkarbeit zu investieren.

## Introduction

In Switzerland, the medical training system is regulated by the state and medical training organisations. The Swiss Institute for Medical and Continuing Education (SIWF) is the central point of contact for all doctors, institutions and authorities. In cooperation with the respective professional associations, the SIWF issues a detailed specialist medical training programme for each field of medicine. Hospitals and practices can apply to the SIWF for recognition as an official training institution [1].

As regional networks of recognized training institutions, consortium structures are intended to improve specialist medical training by promoting knowledge transfer between the participating institutions, coordinating the training pathways of the participating junior doctors and offering targeted supervision and mentoring [2]. While recognized training institutions in Switzerland must be embedded in network structures, young physicians pursuing specialisation are free to choose a specific network and complete all stages of their training within that network, or to undergo training at institutions from different networks. In any case, the minimum requirements of the SIWF must be met, e.g. with regard to the duration of employment in large central or university hospitals (category A), regional hospitals (category B) or training in an outpatient gynaecology/obstetrics practice (separate category). The formal requirements of the SIWF are as follows: “Each training institution is affiliated with a training network. A training network consists of at least one training institution in category A and at least one in category B” [3]. 

However, to ensure that network structures do not exist only on paper, coordination, organisational and communication tasks must be performed on an ongoing basis [4]. To this end, the cooperating clinics and practices can set up a coordination office to manage and evaluate the rotation of junior doctors in the required sections [5].

Depending on the perspective, different goals are formulated for medical training in network structures. On the one hand, the aim is to support young physicians in their professional advancement; on the other hand, in times of the much-discussed medical workforce shortage, the aim is to attract urgently needed young doctors and then retain them in a specific region in the long term [6]. In Switzerland, candidates with medical degrees from foreign universities can also be admitted to specialist medical training. The body responsible for recognising medical diplomas from abroad is the Medical Professions Commission (MEBEKO) of the Federal Office of Public Health (FOPH) [7]. The procedure is clearly regulated and depends on various factors, such as the origin of the degree and individual qualifications [8].

Network structures offer the participating training institutions the prospect of recruiting applicants who are particularly good matches for a position due to the candidates’ individual statuses in the training pathway, for example, if they have already completed a training period at an institution within the network. Since the so-called a-year often proves to be a bottleneck in the specialist medical training pathway [9], network structures give participating category B hospitals the advantage of making it easier for their junior doctors to access a training period in a category A hospital. Outpatient gynaecology/obstetrics practices affiliated with the network can benefit by recruiting experienced junior doctors, who are particularly cost-effective to employ. At the same time, this allows long-term relationships to be established, paving the way for young physicians to later join or buy out existing outpatient gynaecology/obstetrics practices. 

For junior doctors, specialist medical training within network structures can be attractive for various reasons. For example, it eliminates the need to organise the individual stages of training and the associated separate applications to different institutions. If the rotation sequence is determined at the beginning of the training and adhered to throughout, networks offer a realistic opportunity to complete the compulsory training segments prescribed by the training regulations in the minimum time and to thus complete the training quickly [10]. 

However, given the current labour market situation with a shortage of qualified healthcare practitioners [11], [12], it is conceivable that the planning security promised by the training networks over several years can also be achieved by young doctors without joining a network. In fact, the actual or feared restriction of their right to have a say in the selection of positions may even be perceived as a fundamental disadvantage of networks [4]. It should be noted here that the younger generation today starts their working life with different expectations than in the past [13]: Prospective specialists tend to be more selective about their employer and their work conditions. This reflects changing values and expectations with regard to work, leisure time, self-determination, career and family [12], meaning that the attractiveness of networks does not depend solely on purely professional factors. In addition to the assurance of a minimum period of employment in a central or university hospital (known as the A-year), the guarantee of the required catalogue of operations and special training opportunities to improve clinical skills, work-life-balance considerations and the possibility of part-time work also play an important role [9].

### Setting and working methods of the analysed network

The Cantonal Hospital of St. Gallen (*Kantonsspital St.Gallen*: KSSG) is located in the border region with Austria, Liechtenstein and Germany and is divided into five specialist areas: gynaecology and gynaecological oncology, urogynaecology, foetal-maternal medicine and obstetrics, endocrinology and reproductive medicine, and neonatology. Neonatological care is provided in cooperation with the local children’s hospital. At the women’s clinic, more than 2,600 gynaecological procedures (including major tumour surgery, mainly endoscopic) and more than 2,100 births are performed annually (as of 2023), with a high proportion of high-risk pregnancies. KSSG is authorised to provide specialist training in gynaecology and obstetrics (category A) as well as training in the key areas of surgical gynaecology and obstetrics, foetal-maternal medicine, gynaecological oncology, urogynaecology, reproductive medicine and gynaecological endocrinology. 

The women’s clinic at KSSG has joined forces with other clinics in eastern Switzerland to form the “training network for gynaecology and obstetrics in Eastern Switzerland”, whereby structured training to become a specialist in gynaecology and obstetrics usually begins in one of the regional hospitals (category B). After two to three years, junior doctors then transfer to a category A hospital in the network. Prospective specialists also have the opportunity to gain professional experience in designated outpatient gynaecology/obstetrics practices, with a maximum of 12 months (as things stand at present) counting towards their training. In each category, there are institutions that offer specialisation training on a part-time basis. As junior doctors are not obliged to join a training network, almost every participating hospital and almost every outpatient practice also trains junior doctors who are not affiliated with the network.

Between 2012 and 2022, between 10 and 12 hospitals, including two category A hospitals, participated in the network. Since 2014, the network has also included outpatient gynaecology/obstetrics practices. The number of community-based doctors involved in the training network has grown steadily since then to 10 practices (as of 2023). Geographically, the participating training hospitals and outpatient practices are located in the cantons of Appenzell Ausserrhoden, Glarus, Graubünden, St. Gallen and Thurgau.

The coordination office of the Training Network for Gynaecology and Obstetrics in Eastern Switzerland is seated in KSSG’s women’s clinic. It provides information about the network to applicants as well as to enquiring hospitals and practices and manages the network agreements that govern the cooperation between the participating institutions. The contracts stipulate, among other things, that the training institutions are obliged to participate in the biannual network meetings and to comply with the fee schedule. Fees for participation in the network are only charged to the training institutions; the services of the network are free of charge for junior doctors. KSSG plans, conducts and records minutes of the biannual meetings. 

If medical students and junior doctors are looking for an entry-level or continuing training position in the network, they have the opportunity to introduce themselves to the institutions in person at the network meetings or to have the coordination office distribute their application materials by email to all network institutions. Applications received by the coordination office can thus lead to employment as a junior doctor not only at KSSG, but also at another training institution in the network. 

If an application does not result in employment at the KSSG women’s clinic, applicants may give consent for the coordination office to forward application materials to all or to specified training institutions in the network. If hospitals or practices have interest and suitable vacancies, they can contact the applicants immediately and directly. Alternatively, applicants also have the option of introducing themselves in person at network meetings. As these only take place twice a year, this alternative is particularly suitable for applications with long lead times to the desired start date. If candidates are suitable and there are vacancies, they receive an initial offer from the network, which usually comprises the first two years of specialist training in a category B hospital and one year in a category A hospital. The remaining training period until the specialist examination can then be planned during the biannual network meetings. During the COVID-19 pandemic, the meetings and selection interviews were conducted exclusively online.

### Goals and questions

The aim of this quantitative study was to use the application materials and documented career paths of junior doctors to describe the functioning and significance of a network in the context of medical training and to draw conclusions for practical application.

Specifically, the following questions were asked:

- How can the applicants be described in terms of socio-demographic characteristics?
- What level of training did the applicants have at the time of their application to KSSG?
- What career path did the applicants take before applying to KSSG?
- What percentage of applications received by KSSG were sent to other training institutions in the network?
- For how many junior doctors was the career path planned and evaluated in the half-yearly network meetings?
- How many junior doctors whose career paths were planned and evaluated in the biannual network meetings obtained full specialist qualification during this period?

## Method

The study is based on the written application materials addressed to the women’s clinic of KSSG. This is a census survey. All applications received by post or email between 1 January 2012 and 31 December 2022 *for a position as a junior doctor for specialist training in gynaecology and obstetrics* were reviewed, coded according to predefined criteria and then evaluated anonymously. In the case of multiple applications from the same person, the date of first receipt of the documents was entered in the evaluation. The data on the successfully completed specialist examinations of the young physicians is based on the corresponding feedback from the participating institutions during the biannual network meetings.

Of the original 427 applications, 12 had to be excluded from further analysis because two applicants explicitly requested that their documents be returned and the subsequent application process of 10 applicants could no longer be fully traced. The statistical analysis (frequency counts, mean values, minimum, maximum, mode) was performed using IBM SPSS Statistics 25. An ethics vote was not obtained because the analysis did not include any health-related data. Only employees who already had access to the application documents in their daily work were given access to the non-anonymised original data. 

## Results

### Sample description

A total of 415 applications were analysed with regard to the research questions. On average, 38 applications (minimum=26, maximum=51) per calendar year were received between 2012 and 2022 for positions as junior doctors pursuing training in gynaecology and obstetrics at the KSSG women’s clinic.

### General description of applicants

**Figure 1:**
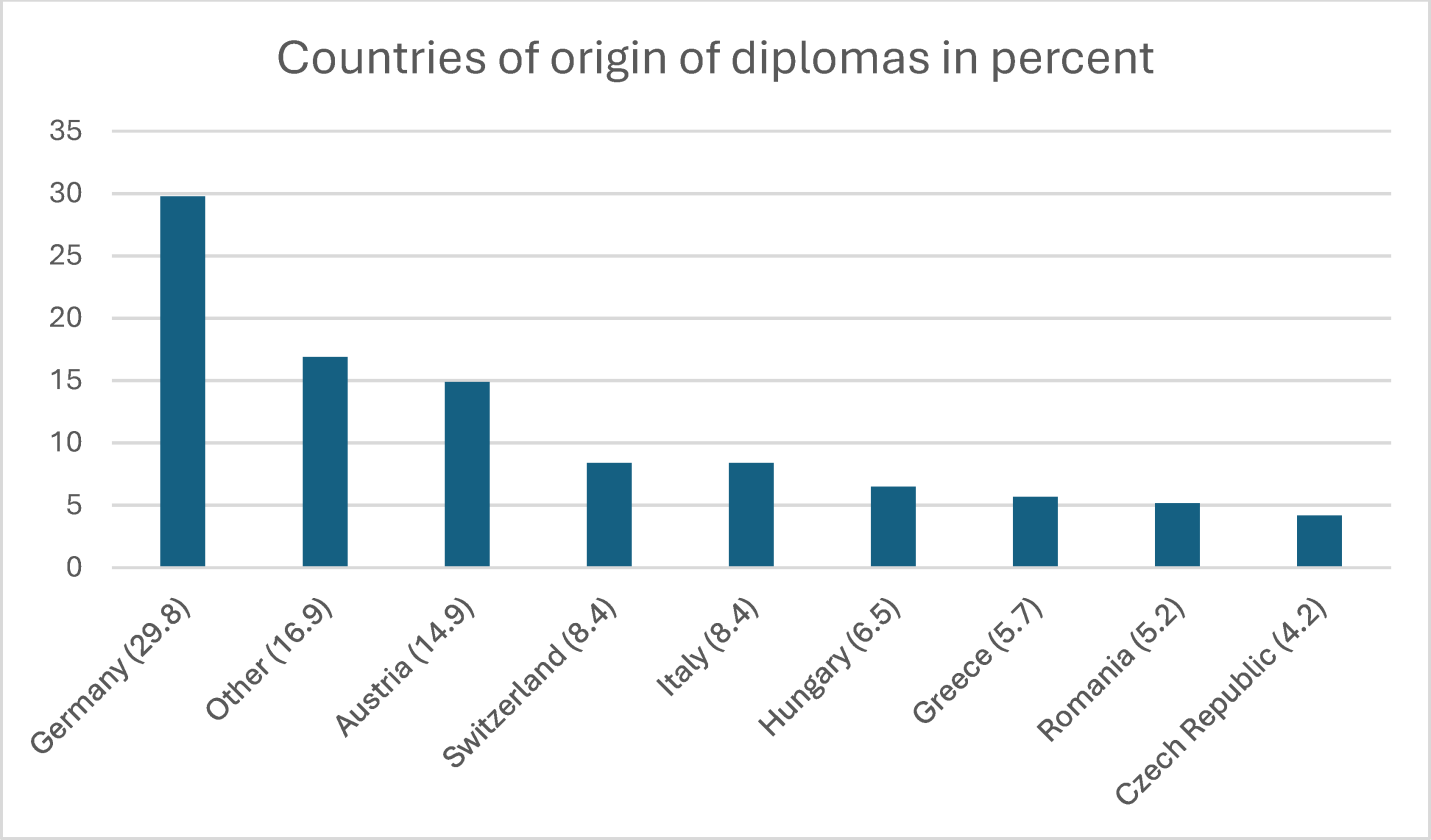
Medical degrees by country of origin (n=403), countries listed under “Other” each account for a maximum of 1.5 percent

Between 2012 and 2022, 336 women (81.0 percent) and 79 men (19.0 percent) applied for a position as a junior doctor. The average age was 29.8 years (minimum 23 years, maximum 56 years, mode 28 years). 10.6 percent of applicants had a Swiss passport. Overall, the candidates held medical degrees from 37 different countries. Table 1 (Tab. 1) and figure 1 (Fig. 1) show the countries of origin of the applicants and their degrees in detail. Looking at individual calendar years, we see that in 2012 (2022), 26 (36) applications were received, with 3.8 (11.1) percent holding a Swiss diploma. 46.2 (27.8) percent had obtained their medical licensing examination in Germany and 30.8 (5.6) percent in Austria. 

### Level of training and career path of applicants

27.1 percent of candidates applied immediately after passing their medical licensing examinations, and 3.0 percent before completing their studies (on average 3 years after passing their medical licensing examinations, minimum 4 years before completing their studies, maximum 25 years after). Before applying to the women’s clinic at KSSG, 58.8 percent of candidates had already worked as junior doctors in the field of gynaecology and obstetrics at other locations in Switzerland and abroad, and 18.1 percent at a clinic belonging to the Eastern Switzerland network. 48.9 percent of applicants stated in their documents that they had previously worked as junior doctors in any field within Switzerland. 43.1 percent had already established initial professional contacts in Switzerland before completing their medical studies and were employed in Switzerland as part of an internship in any medical field.

#### How the training network works

**Figure 2:**
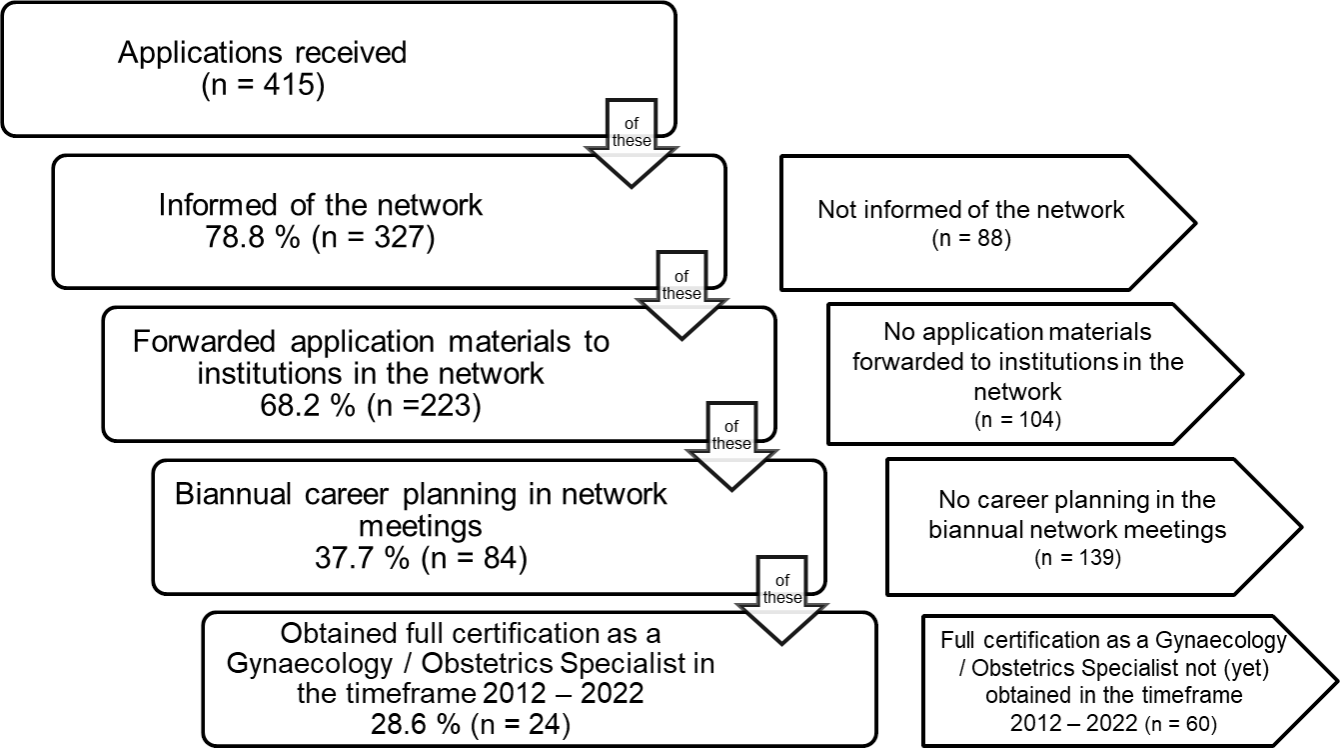
Operation of the network – quantitative analysis

The coordination office informed 78.8 percent (n=327) of applicants about the possibility of training within the training network for gynaecology and obstetrics in Eastern Switzerland. Of these, 68.2 percent (n=223) subsequently agreed to have their application documents sent to all or selected training institutions in the network. For 37.7 percent (n=84) of these applicants, their individual career path was then planned, managed and evaluated at the network’s biannual meetings. Of these, 28.6 percent (n=24) obtained their qualification as specialists in gynaecology and obstetrics during the period under review without having left the network. Figure 2 (Fig. 2) shows how the network's coordination office works, quantified in terms of applications received at the Women’s Clinic of KSSG for positions as junior doctors between 2012 and 2022.

## Discussion

This quantitative study of the working methods of the training network for gynaecology and obstetrics in Eastern Switzerland shows that network structures can be used to increase the reach of applications. The majority (78.8 percent) of applicants to the KSSG women’s clinic were informed by the coordination office about the possibility of medical training in the training network for gynaecology and obstetrics in Eastern Switzerland. Only about one in five applications (21.2 percent) was processed independently of the network at the women’s clinic of the hospital in question. The reasons for this were that these applicants had either already ruled out placement or planning via the network in their cover letter or were considered only for a position at KSSG. Further studies with a qualitative research design could clarify whether and through which activities of the coordination office it is easier for the network partners to fill positions.

The fact that applicants explicitly do not want their application documents to be forwarded or passed on within the network structures may be perceived as “rebellious” [14] by the older generation, but it shows how things have changed. Earlier generations had to be grateful to get a job and therefore accepted even unacceptable conditions [15]. Today, junior doctors no longer have to make themselves attractive to hospitals; instead, hospitals are the ones trying to appeal to young physicians [16]. Young doctors will likely be put off by overly rigid network regulations that restrict their perceived decision-making freedom without providing any distinct and clearly communicated added value. Such structures primarily serve to ensure planning security for the hospitals and practices involved and are unattractive to the rising generation of physicians [4].

Between 2012 and 2022, 81.0 percent of women and 19.0 percent of men applied for a position as a junior doctor at KSSG’s women’s clinic. The high proportion of women is not surprising, as it is also evident in the later career phase: after the specialist examination, gynaecology and obstetrics has the highest proportion of women of all medical specialties in Switzerland (69.6 percent), followed by paediatrics and adolescent medicine (68.4 percent) [17].

As early as 2016, the FMH (Foederatio Medicorum Helveticorum, Swiss Medical Association) attested that foreign-born specialists make a significant contribution to maintaining medical care for the population in Switzerland [18]. In 2022, the authors of the “FMH medical statistics” again described Switzerland’s heavy dependence on doctors trained abroad. At that time, 39.5 percent of *practising doctors* had a *foreign degree*. The majority of medical professionals with a foreign degree came from Germany (51.0 percent), followed by Italy (9.4 percent), France (7.2 percent) and Austria (6.0 percent) [19].

In comparison, the proportion of *applicants with foreign qualifications* at the hospital considered in this study was significantly higher in 2022, at 88.9 percent. This may be related to the geographical location in a border region or questions of the attractiveness of eastern Switzerland as a location for foreign applicants compared to domestic applicants, but it may also simply be due to the fact that an application is not synonymous with a subsequent professional activity in Switzerland, which depends on many other factors. Foreign doctors who come to Switzerland temporarily for training may apply for a position as a junior doctor, but in the long term they may return to their countries of origin or move to other countries [20], [21]. Whether foreign workers who come to Switzerland for specialist medical training stay in the long term depends on personal factors and on working conditions in the Swiss healthcare system relative to other countries [22].

Overall, the applicants in this study held medical degrees from 37 different countries. Even though over 90 percent of applicants had passed their medical licensing examinations in one of the 27 EU member states or in Switzerland, this shows how international the young generation of doctors is today [14], [23]. Since Switzerland is also known for its high cost of living, financial incentives alone are unlikely to be a sufficient explanation for the large influx of foreign applications. Other factors, such as the hospital’s location near international borders, the quality of training, the reputation of the Swiss healthcare system or career development opportunities may play a greater role. It is also possible that the network structures are particularly attractive to foreign applicants. Information on the training network for gynaecology and obstetrics in Eastern Switzerland is available on the KSSG women’s clinic website. The organised and structured combination of different training institutions and the facilitated rotation between them are likely to contribute to the fact that training in the network appears particularly appealing to foreign professionals. Not yet familiar with the structures of another country, the tailor-made training offered by a network can give doctors wishing to emigrate the security they need to ultimately take the leap to move to Switzerland from abroad. Further research is needed on the motives that lead young doctors to Switzerland in general and specifically to the medical training network described in this study. Qualitative studies in particular are the method of choice in order to obtain differentiated data and subjective perspectives.

This study describes applicants for specialist training in gynaecology and obstetrics in terms of socio-demographic criteria, their career paths and their willingness to use a specific network offering. It is conceivable that the application structure of other clinics and other training networks differs from the one presented here. In order to assess this, similar studies of other hospitals or training networks in Switzerland would be desirable. This applies even more so to international comparisons: the extent to which the results can be transferred to other countries with different training requirements and structures cannot be assessed on the basis of the data collected. A first step would be, for example, to discuss the topic of “specialist training in network structures” at a professional conference. Despite its local limitations, the scope of this study as a census survey and the long inclusion period can be considered strengths. The “FMH medical statistics” point to Switzerland’s high dependence on foreign countries based on the number of foreign diplomas held by doctors already working in Switzerland [17]. The present study takes up this issue from the timepoint of application for a specialist training position and shows the high mobility of young doctors. 

One limiting factor is that the study is based exclusively on the data available to the network's coordination office, which has been collected continuously since 2012. For example, there is no information on how many applicants actually obtained employment at an institution in the network after the initial distribution of application documents outside KSSG. It is conceivable that although employment was obtained at a network institution, the corresponding feedback was not provided to the coordination office for a variety of reasons, e.g. because the applicants expressly did not want this or because the subsequent training phase was completed at an institution outside the network. Since junior doctors are not obliged to join a specific training network, there are ultimately limits to the traceability of career paths. This could be countered by obtaining the consent of the junior doctors to contact them via their private email addresses and ask them for information about their further career path, but here too, the aspect of the effort involved in networking plays a role, which would have to be invested by the coordination office and the participating institutions in order to ensure complete traceability. 

The present study did not collect data on the reasons why certain applicants expressly reject job placement and career planning within the network. However, clues can be gleaned from earlier surveys: the actual or feared restriction of their right to have a say in job selection may also be perceived by young doctors as a fundamental disadvantage of networks [4].

Of those junior doctors whose career paths were regularly discussed in network meetings, 28.6 percent obtained their specialist qualification in gynaecology and obstetrics during the period under review without having left the network beforehand. This rate appears low, but it is probably too low to be considered proof of the success of the existing network structure, as the coordination office did not have any data on the successful completion of training by candidates who had only completed part of their training in the network and were working at an institution that did not belong to the training network for gynaecology and obstetrics in Eastern Switzerland at the time of their specialist examination. Further research is therefore needed in this area as well. Points of comparison could then be, for example, the completion rates of other networks or the overall completion rates of specialist training in gynaecology and obstetrics in Switzerland, as well as the average time taken to complete the specialist examination.

Last but not least, the significance of the present study is limited by the fact that the results could be distorted by bias on the part of the investigators, as the network's coordination office is organisationally integrated into KSSG.

## Conclusions for practice

Today’s young doctors are mobile and internationally oriented. For training networks, this presents both a challenge and an opportunity. In order to retain young doctors in a particular geographical region in the long term, it is necessary to offer attractive training programmes tailored to their individual needs. For foreign professionals in particular, medical training in network structures could offer special advantages due to the organised and structured combination of different medical training institutions. Networks increase the reach of applications and can support the participating clinics in recruiting medical staff. Due to their regional and long-term orientation, they can also make an important contribution to ensuring high-quality healthcare for the population, especially in rural areas. This requires all parties involved to invest jointly in networking. To ensure that networks do not only exist on paper, coordination, organisation and communication are needed – as well as a good degree of conflict management skills in order to meet the diverse demands.

## Authors’ ORCIDs

- Ellie B. Schmidt: [0000-0002-0564-9496]
- René Hornung: [0000-0003-4564-3678]

## Competing interests

The authors declare that they have no competing interests. 

## Figures and Tables

**Table 1:**
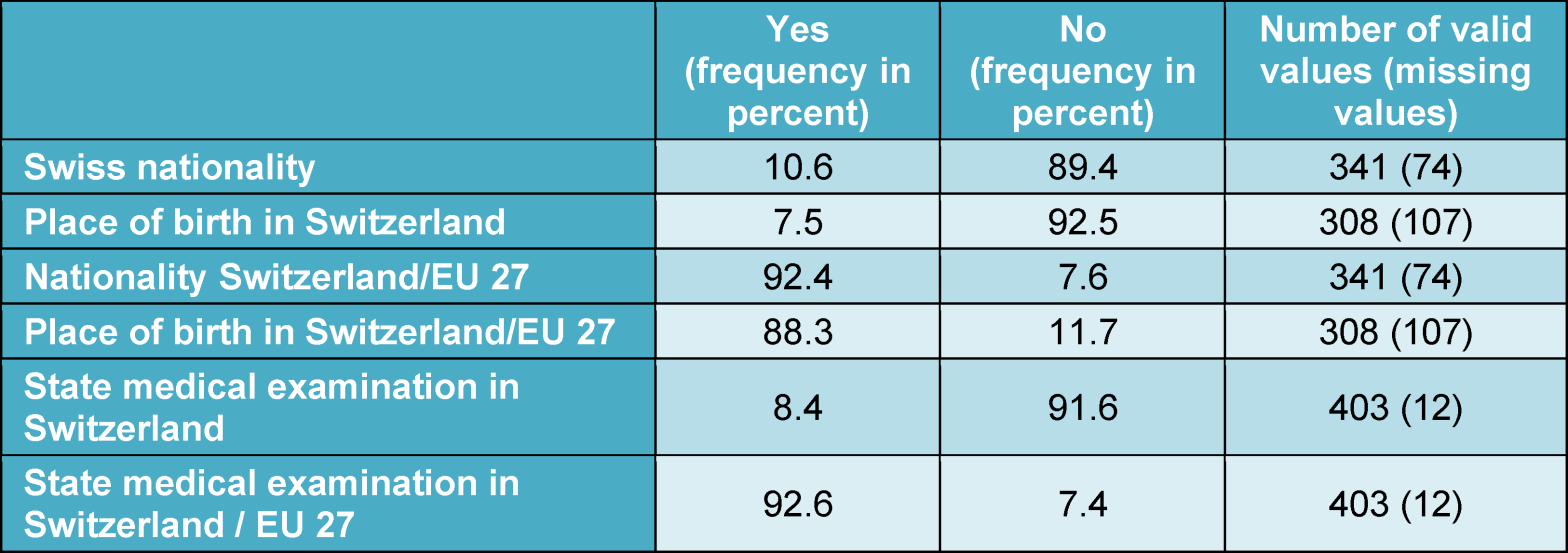
Description of applicants by country of origin (nationality, place of birth, diplomas)
